# 3D printing filament as a second life of waste plastics—a review

**DOI:** 10.1007/s11356-020-10657-8

**Published:** 2020-09-04

**Authors:** Katarzyna Mikula, Dawid Skrzypczak, Grzegorz Izydorczyk, Jolanta Warchoł, Konstantinos Moustakas, Katarzyna Chojnacka, Anna Witek-Krowiak

**Affiliations:** 1 Department of Advanced Material Technologies, Faculty of Chemistry, Wrocław University of Science and Technology, Smoluchowskiego 25, 50-372 Wrocław, Poland; 2 School of Chemical Engineering, National Technical University of Athens, 9 Iroon Polytechniou Str., Zographou Campus, GR-15780 Athens, Greece

**Keywords:** 3D printing, Filament, Recycling, Polymer, Plastic, Waste management, Extruder

## Abstract

In recent times, the issue of plastic recycling has become one of the leading issues of environmental protection and waste management. Polymer materials have been found an application in many areas of daily life and industry. Along with their extended use, the problem of plastic wastes appeared because, after withdrawal from use, they became persistent and noxious wastes. The possibility of reusing polymeric materials gives a possibility of valorization—a second life—and enables effective waste utilization to obtain consumable products. The 3D printing market is a well-growing sector. Printable filaments can be made from a variety of thermoplastic materials, including those from recycling. This paper focuses on a review of the available literature on the production of filaments for 3D printers from recycled polymers as the alternative to present approach of central selective collection of plastics. The possibility of recycling of basic thermoplastic materials and the impact of processing on their physicochemical and mechanical properties were verified (Lanzotti et al. 2019). In addition, commercially available filaments produced from recycled materials and devices which allow self-production of filaments to 3D printing from plastic waste were reviewed.

## Introduction

**Fig. 1:**
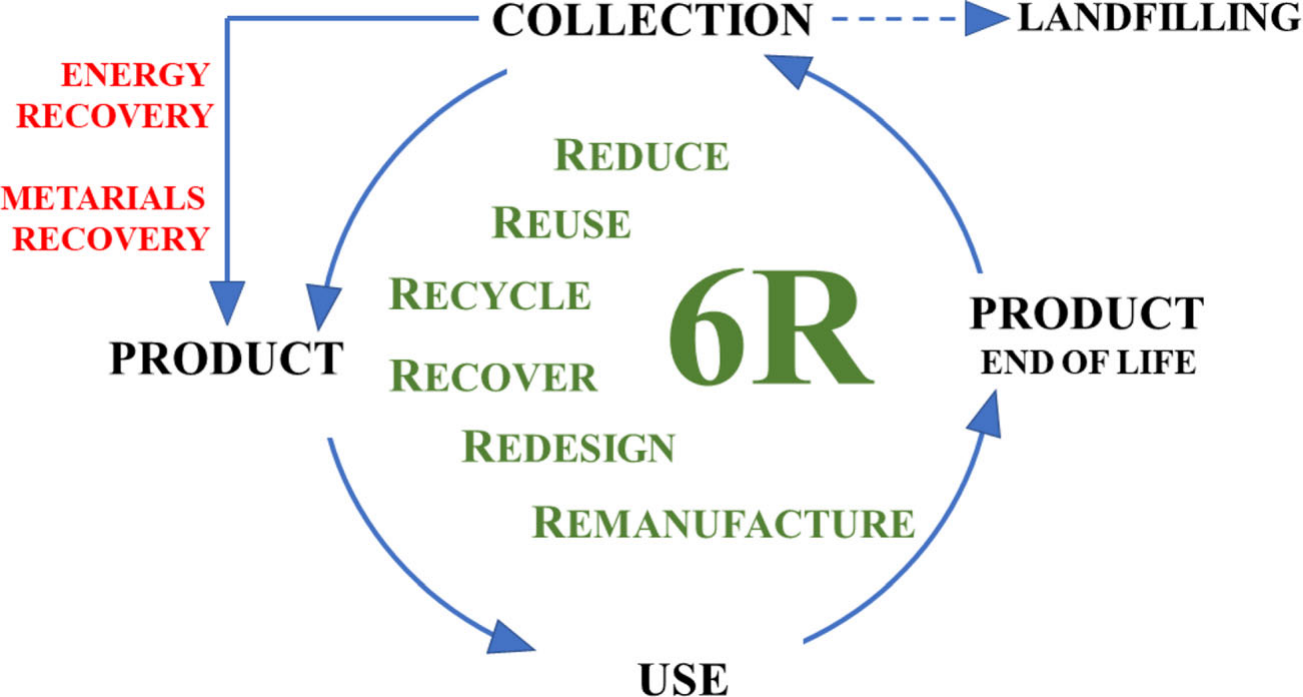
The basic concept of circular economy

Circular economy (CE) concept is a response to environmental and social problems, being a replacement for the previously used linear concept based on the “take–make–dispose” model. Population growth, intensive use of resources, and uncontrolled environmental pollution forced the implementation of another economic closed-loop system, based on the principles of 3Rs: Reduce, Reuse, and Recycle. The broader methodology (6R, Fig. 1) includes additional three approaches: Recover, Redesign, and Remanufacture (Jawahir and Bradley 2016).

Circular economy goals were set for Europe in Circular Economy Package on 2 December 2015 (“EUR-Lex-52015DC0614-EN-EUR-Lex,” accessed 2020.04.09). The CE package lists five priority fields requiring specific action: plastics, wastes from demolition and construction, critical raw materials, food waste, bioproducts and biomass. This plan considers specific key targets, including recycling of municipal wastes at the level of 65% until 2030, recycling of packaging waste at the level of 75% until 2030, and reduction of landfilling to a maximum of 10% until 2030. CE is designed to be a restorative and regenerative system, beneficial to society and economy, allowing for the reduction of natural resources, and waste and environmental pollution minimization, as well as for the recovery of materials and energy (Fig. 1) (Kaur et al. 2018). Recycling is recognized as the most preferred option of waste management for reuse of the materials in order to manufacture new products (Mwanza and Mbohwa 2017). Such practices decrease waste generation and enable material recovery as long as possible. One of the main wastes, which is currently a serious environmental problem, is plastic.

The aim of this work was to review the possibility of reusing polymeric materials for 3D printing. The attention was paid to the recycling potential, existing commercial solutions, and programs related to the promotion of the idea of reuse of waste materials. The work also included possible changes in the polymer material, which may occur during subsequent extrusions.

## Plastic waste management

Plastics have appeared in our daily lives around 100 years ago; still they are indispensable materials with various properties and applications used at home, at work, traveling, or in their spare time. Plastics are extremely versatile materials, so that the possibilities of their application are virtually limitless. Their unquestionable advantage is the high mechanical strength, low density, low weight, easy processing, and low cost (Mwanza and Mbohwa 2017). Due to these features, plastics have been found applications in the production of packaging, automotive industry, electricity, construction, and transport, as well as in medicine, agriculture, or other areas. The ubiquitous plastic is a source of huge amounts of waste, the management of which is a serious problem. Global production of plastics in 2018 amounted to 359 million metric tons (in EU 61.8 million metric tons) (www.plasticeurope.org, accessed 2020.04.09). It is predicted that this number will double over the next 20 years.

In many countries, plastic waste is not managed and goes to landfills. Landfill space is limited and the amount of plastic stored is growing rapidly every year. Tighter regulations on waste management need to enforce recovery of materials and energy so as to meet the requirements of the circular economy project. In EU, 75.1% of plastic waste was processed (32.5% recycling and 42.6% energy recovery) while 24.9% still was landfilled (www.plasticeurope.org, accessed 2020.04.09). There are many methods to manage the growing amount of plastic waste, including primary recycling (re-extrusion), mechanical recycling, and chemical reuse or the use of thermal methods that generate energy (combustion, pyrolysis, gasification) (Al-Salem et al. 2009). Primary recycling allows the recovery of uncontaminated polymer residues with parameters corresponding to the starting material. It can be applied to residues that have not been used in the production process, e.g., in the extrusion, which is popular in most production centers (Singh et al. 2017a). Secondary recycling additionally uses materials that may contain contamination. These impurities are removed during conversion, after preliminary shredding. Such material is successively milled and granulated and becomes an input to plastic processing, but is usually of lower quality than that of primary recycling. Chemical recycling includes chemical processes that convert plastics into compounds that can be reused for production, mainly in depolymerization processes (solvolysis) (Singh et al. 2017a). Methods of energy recovery from polymeric materials are the least environmentally beneficial option, but the energy content of plastics is significant; they are highly efficient energy sources with similar calorific value as fuel oil (average 42 MJ/kg) (Kumar et al. 2011). However, it is necessary to continuously monitor emissions from such processes, as they can generate many organic pollutants such as dioxins (Ragaert et al. 2017). Plastics generally cannot be decomposed by microorganisms because bacteria have not developed enzymes that enable the biological decomposition of these materials (Shah et al. 2008). However, biodegradable materials that degrade under the influence of the environment are designed, such as various types of polyesters, including polylactide (PLA) or polycaprolactone (PCL) (Shah et al. 2008), which are used in the production of 3D printing filaments.

Environmental hazards resulting from the pyrolysis of plastics and the under-degradability of polymers force them to be processed. Landfilling is only a temporary solution. Given the continuous production of plastics and the very low degree of processing, a new solution is necessary. Of course, the best waste management strategy is to prevent their generation. Unfortunately, many factors, such as convenience, consumer lifestyle, advantage of characteristics, and production costs over glass and metal packaging, make plastics abundant in the environment and persistent. A waste-free economy is only theory that waits to be implemented. Therefore, cost-effective plastic processing technologies are urgently needed. In addition to the available technologies, 3D printing using waste polymers is a new, potential solution with the highest possible degree of future implementation.

## Commercial polymers for 3D printing

3D printing market is one of the fastest growing sectors. It is expected that by 2021 it will see over 23% market growth comparing to 2016, reaching over 10 mld USD (Shah et al. 2019). 3D printing is a relatively new technology that has become very popular in the last few years. Simplicity and low costs have contributed to the fact that it is primarily used in prototyping and small-scale productions. In recent years, the use of 3D printing has become more popular in various industrial sectors, with the aerospace, military, automotive, medical, and construction industries increasingly taking advantage of it (Shah et al. 2019).

The filaments used in 3D printing are primarily thermoplastics. The most popular are acrylonitrile butadiene styrene (ABS) and polylactic acid (PLA) (Anderson 2017). The remaining group of materials are polycaprolactone (PCL), polycarbonate (PC), polystyrene (PS), polyetherimide (PEI), polyetheretherketone (PEEK), and various types of polyethylene (PE), including LDPE (low-density PE), LLDPE (linear low-density PE), and HDPE (high-density PE). These types of materials are commonly used to print automotive components, surgical instruments, prototypes, various types of packaging, small garden architecture, toys, and many other products that are in everyday use.

Despite the advantages, 3D printing generates large amounts of waste, which are the result of failed prints or rejected support structures. What is more, the ability to create components without machining or tools causes that many prints are used as disposable prototypes. The number of thermoplastic prints is constantly growing with the development of additive technology, so there is the problem of waste management. The solution may be filaments obtained from the recycling of plastics. Filaments used in 3D printing are most often formed in the extrusion process, by inserting a granulate or polymer powder into the extruder, which, under the influence of temperature, is transformed into a homogeneous material in the form of the line with defined parameters (standardized diameter, adapted to the size of the printer element). An increasing number of companies offer filaments from recycled PLA or ABS. Unfortunately, so far there is not much information about the mechanical properties of recycled filaments. They have a crucial influence on print quality. Thus, investigating these properties of recycled filaments and comparing them with virgin material will provide the basic knowledge necessary for the further development of 3D printing technology (Anderson 2017).

## Recycled polymers for 3D printing

The global production of plastic-based goods has increased significantly in recent years. According to PEMRG (*Plastics Europe Market Research Group*), global plastic production in 2019 amounted to 359 million tons, of which 51% is in Asian countries and 17% in Europe. Each year, 4% of world oil production (1.3 billion barrels a year) is used to produce virgin plastics (Singh et al. 2017b). The polymers used for production are mostly not degraded and remain in the landscape for many hundreds of years (Gu and Ozbakkaloglu 2016). For this reason, environmental pollution associated with this type of waste is a serious problem. Data shows that up to 90% of plastics could be reused. Currently as much as 80% of plastic waste is in landfill and only a few percent is recycled. The biggest problem is plastics made from HDPE, LDPE, PP, and PVC, which are largely used by manufacturers and which are landfilled with greenhouse gas emissions (Aboulkas et al. 2010). A much smaller global problem is PLA-related waste, whose natural origin does not have such a strong impact on the environment. Unfortunately, goods made of this material are less durable mechanically, which in turn discourages potential manufacturers from using them more often. The main limitation related to the reuse of the material is the problem of losing the properties after recycling several times. Additionally, stability loss is observed, which in turn may adversely affect human health (Lithner et al. 2011).

The 3D printing technology allows more possibilities to create complex structures on a small scale. The evolving technology also poses risks associated with the generation of more plastic. However, the data show that at present, 3D printing waste is not such a big problem and the technology itself can be used to combat the growing amount of post-production waste (Cruz Sanchez et al. 2017). The recycling process of polymeric materials for 3D printing is based on a number of activities such as selective material separation, decontamination and purification, grinding, re-melting, and extrusion. The main obstacles resulting from this process are logistical and economic aspects. The analysis shows no economic benefits from the recycling of materials, and the cost of the recycled product depends on the market price of the originally manufactured filament (Hopewell et al. 2009). However, given the increasing environmental restrictions and recycling of plastic waste, this could be a potential solution, despite the lack of clear economic profitability.

**Fig. 2:**
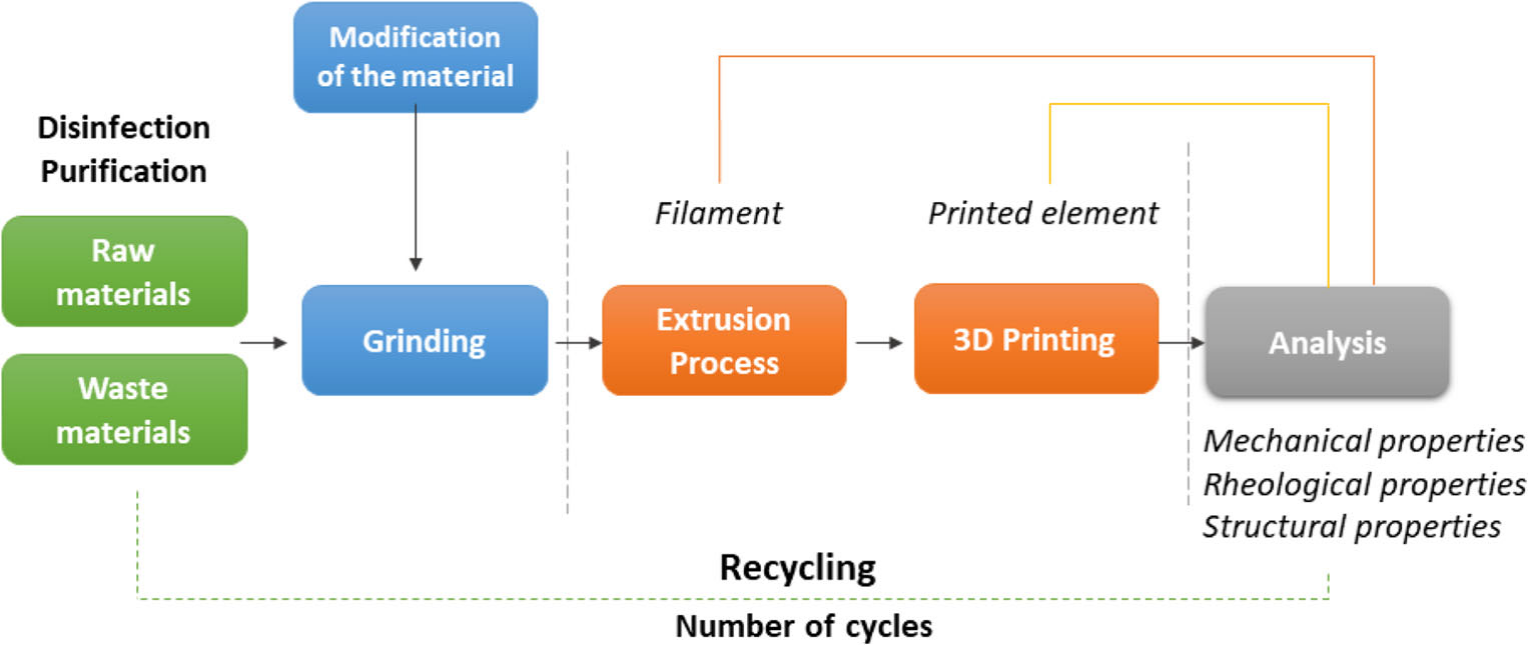
Manufacture of filament from waste materials

Globally, seven groups of plastics are currently being recycled: polypropylene (PP), polyvinyl chloride (PVC), high- and low-density polyethylene (HDPE, LDPE), polystyrene (PS), polyethylene terephthalate (PET), and the “other” group, mainly ABS and polycarbonate (PC). According to the literature, all of the above groups have been investigated for their potential re-use in the form of a 3D printing filaments (Table 1). However, the largest part of the research on the possibility of using plastics for 3D printing is the natural origin of PLA. The influence of multiple material recycling was studied (Zenkiewicz et al. 2009; Anderson 2017), as well as the possibility of introducing an additional strengthening component (Pillin et al. 2008; Gkartzou et al. 2017; Zhao et al. 2018b). Based on the available research, a general scheme of waste recycling for 3D printing was created (Fig. 2).

**Table 1:** Reuse of waste from different origins to produce 3D printing filaments

Materials	Origin	Additives	References
PLA	PLA type 4043D (NatureWorks)	-	(Cruz Sanchez et al. 2017) (Cruz et al. 2015)
PLA	INGEO 2003D (Natureworks LLC)	Craft lignin	(Gkartzou et al. 2017)
PLA	Filament (FLASHFORGE Corp Japan)	Carbon fiber reinforced (CFR)	(Tian et al. 2017)
PLA	Type 2002D (Natureworks USA)	-	(Zenkiewicz et al. 2009)
PLA	Broken PLA parts fabricated by 3D printing	Polydopamine (PDA)	(Zhao et al. 2018b)
PLA	PLLA L9000 (Biomer)	Tropolone, p-benzoquinone hydroquinone	(Pillin et al. 2008)
PLA	Unknown source	-	(Anderson 2017)
PLA	Commercial grade (Ingeo 2003D, Natureworks)	-	(Beltrán et al. 2018)
PLA/RPLA	Industrial waste mix	-	(Cisneros-López et al. 2020)
ABS	ABS–post-consumer	-	(Woern et al. 2018)
ABS	Virgin pellet material/failed-redundant 3D prints	-	(Mohammed et al. 2017)
PET	Water bottles	Biochar	(Idrees et al. 2018)
PET	Water bottles Drink bottles Salad containers	-	(Zander et al. 2018)
PET	Recycled material (Gruppo Mossi & Ghisolfi, Brazil)	Lignocellulosic	(Santos et al. 2018)
PET	Unknown source	-	(Exconde et al. 2019)
HDPE	HD50MA180 (Reliance Polymers)	-	(Singh et al. 2018)
HDPE	Unknown source	-	(Baechler et al. 2013)
HDPE	Detergent containers Shampoo bottles Household bottles Milk bottles	-	(Chong et al. 2017)
PP	Granules of pre-consumer recycled PP (Astron, Auckland, New Zealand)	Hemp fiber Harakeke fiber MAPP (maleated polypropylene) Recycled gypsum	(Stoof and Pickering 2018)
PC	Electronic waste from printers	-	(Sahajwalla and Gaikwad 2018)
PET, PE, PP, Fim, Mix	Plastics products–household	-	(Brouwer et al. 2018)
LDPE LLDPE HDPE	LDPE, CA 8200 LLDPE, LE 1000 HDPE,CB 9600 (Borealis OY)	-	(Andersson et al. 2004)
PC/ABS (CS)	PC (MD-1500) ABS (PA-717C) CS (CELEX 5200HF)	-	(Chiu et al. 2018)
PLA ABS PET PP	Failed 3D prints NorthWest polymers CiorC McDonnough plastics	-	(Woern et al. 2018)
LLDPE LDPE	Meal bags (MRE)	-	(Hart et al. 2018)
PP HDPE	Postconsumer hard plastics Plastic bags (DA.IA Technology, Taiwan)	Iron Silicon Chromium Aluminum (nano-crystalline powders)	(Pan et al. 2018)
HDPE	Unknown source	Zirconium oxide	(Singh et al. 2019b)
Mixture of polymers PET, PP, PS	Raw materials from recycling bins	-	(Zander et al. 2019)

In the first stage, the material is segregated and washed, and then the plastic is ground. In the next stage, the ground material is extruded at high temperature (the temperature is set depending on the polymer type). The prepared filament is inserted into the 3D printer. The printed element is subjected to analysis (mechanical, rheological, and structural properties). The tested sample is again milled (Zenkiewicz et al. 2009). In case of material modification, an additional stage appears: in the first case, an additional component and a binder (e.g., silicone oil) are added to the mixed material and subsequently extruded (Pan et al. 2018); in the second case, the ground element is dissolved in an organic solvent with an additional reinforcing component, the solvent is evaporated, and the ground material is extruded.

## The impact of recycling on the material properties

Shear stress, temperature, and oxygen occurring during extrusion degrade polymers. The process takes place not only in polymers sensitive to these factors (PLA) but also in polymers that are relatively resistant (PE) (Anderson 2017). The change of the physical properties of the polymer significantly influences the obtaining of high-quality extrusion products. Multiple extrusion of polymers has a strong influence on their change in viscosity, molecular weight, and breaking strength. Changes in properties are generated by factors such as temperature, but also by the amount of extrusion of one material (Zenkiewicz et al. 2009).

### Mechanical properties

Polylactic acid (PLA) and acrylonitrile butadiene styrene (ABS) are the most popular filament materials among the thermoplastics which are currently available for 3D printing. The costs of commercial filaments are up to 200 times higher than those of raw plastics (Cruz Sanchez et al. 2017), though their thermo-mechanical recycling would significantly contribute to the reduction of 3D printing cost. ABS is produced from oil and used for a variety of durable goods despite being toxic. In contrast, PLA is bio-based, biodegradable, and biocompatible polymer (Duval 2014). Its important drawback is high sensitivity to elevated temperature (~ 200 °C) which induces degradation of the macromolecular structure. Pillin et al. (2008) identified a decrease of the PLA chain length with the number of injection cycles. Shorter polymer chains can effectively reorganize themselves into more ordered crystals. It corresponds with significant increase of the melt flow rate. The tensile strength and tensile strain at break were found to be slightly diminished as compared with the tensile stress at break. The reduction of the later was above 8%, with the largest decrease after the first extrusion (4%) (Zenkiewicz et al. 2009). According to Pillin et al. (2008), the decrease of stress at break is attributed to a lower cohesion, while the decrease of strain at break is associated with the decrease of the chain length and the increase of the crystallinity degree. On the other hand, the content of new carbonyl compounds after recycling is marginal.

The heating of PLA causes a lowering of the cold crystallization temperature and diminishing of the melting point. Most authors reported a marginal decrease in the molecular weight subjected to one reprocessing step (Badia et al. 2012; Chariyachotilert et al. 2012). Degradation increases up to 30% after 3 cycles and 60% after 7 cycles (Pillin et al. 2008; Brüster et al. 2016). The TG analysis confirmed successive weight loss along with the extrusion cycles. It starts around 320 °C and most of weight evaporates after 600 °C. Zenkiewicz et al. (2009) claimed that the thermo-mechanical recycling promotes transesterification in the presence of free radicals. Furthermore, the authors identified increase in transmission of water vapor up to 40% and oxygen up to 20% with the number of the extrusion cycles. Both activating agents are considered precursors of free radical reactions. Other suppositions of degradation could result from hydrolysis and transesterification with residual catalysts. The reduction of the intrinsic viscosity during hydrolytic degradation is minor without the washing step of the material. In opposite, significant decrease in the viscosity observed in the case of washed PLA wastes, results from influence of high temperature and shear stress during the polymer reprocessing. The degradation during the accelerated ageing can also contribute to this process (Beltrán et al. 2018) An addition of oxidative stabilizers (quinone) and residual catalyst stabilizers (tropolone) to a neat polymer significantly limits the rheological degradation.

The data obtained clearly identified that recycling reduces the mechanical strength of PLA. To overcome this problem, it is possible to coat the recycled polymer filament with a polydopamine (PDA). PDA adhesive aqueous solution is adsorbed on hydrophobic finger-like surface of PLA by development cohesive strength through self-polymerization. The PDA coated polymer is thermal-stable up to 200 °C. It possesses higher tensile strength and strain at break, and its surface exhibits higher adhesion than uncoated PLA (Zhao et al. 2018b).

Anderson (2017) proposed direct recycling of the utilized PLA filament through its ground up and re-extrusion into 3D printing filament. After two extrusion cycles and one 3D printing process, the material retains similar diameter and surface finish as the original one, while its mechanical properties exhibit slight deteriorations. Unexpected reduction in viscosity, attributed to chain scission during recycling, is the main drawback that prohibits subjection of the utilized PLA filament to further 3D printing (Zhao et al. 2018a, b). The identified rearrangement into lamellar structure indicates randomization of the polymer chains as a consequence of reduced molecular weight. According to Beltran et al., the increment of crystallinity and the number and average size of pinholes in twice recycled PLA filament do not result from repeated extrusion but rather from 3D printing. The thermal process causes shortening of polymers chains, which are able to crystallize more easily with a higher population of crystals (Beltrán et al. 2016). The addition of virgin PLA to the recycled and shredded PLA filament significantly improved the viscosities of the blend as well as increased its mechanical and thermal properties (Zhao et al. 2018a, b). Such a remediation strongly contributes to the closed-looped recycling of PLA filament, which can be done in a bench top machine at home (Di Maria et al. 2018).

Very promising distributed recycling of PLA and ABS wastes is fabrication of 3D printing filament by usage of recyclebot (domestic plastic extruder) combined with an open source self-replicating 3D printer (Baechler et al. 2013). The computer wastes were mechanically cleaned to eliminate impurities which could influence filament consistency and cause clogging in the nozzle of the 3D printer. The heating temperature was maintained below the decomposition of molecule structures and over the glass transition temperature to correlate the properties of the printout with ABS degradation (Zhong and Pearce 2018). In order to avoid the formation of bubbles on the surface of the filament, the ABS material needs to be dry and crushed. Cruz Sanchez et al. (2017) presented a comprehensive analysis of the PLA filament degradation in 5 reprocessing cycles. The data obtained showed a considerable reduction in tensile strength and breaking strength, and nominal deformation at break. The identified result of material decomposition relates to the decrease in crystallinity, viscosity, and molecular composition. It is claimed that the degradation mechanism involves the following: (1) formation of oligomers (hydroxyl and carboxyl); (2) esterification; (3) intermolecular transesterification, including interchanging of ester units between different chains; (4) thermo-oxidation; and (5) micro-compounding process. Moreover, the printing process itself also has an effect on the filament degradation. Irregular cooling and heating cycles result in accumulation of stress in the built part, and thus consequently affect mesostructured and fiber-to-fiber bond strength (Tymrak et al. 2014). Among other factors one can mention growth of the neck between filaments and layers, randomization of the polymer chains on the contact surface, molecular diffusion, and the internal defects (e.g., voids and staircase effect) applied to the material during printing (Sun et al. 2008).

### Additives for polymer manufacturing

To solve the problem of environmental pollution and to find the alternative for shrinking post-petroleum plastic sources, many works have been conducted to obtain a new generation of 3D printing materials. The use of various types of additives with increasing molecular weight and improving mechanical properties of recycled polymer has been widely studied. Research has been conducted on both additives, extending polymer chains and additives in the form of peroxides, which allow the formation of free radicals (molecular weight increase, cross-linking agent).

The effect of the additive lignin on the morphology, mechanical, and thermal parameters of recycled PLA was examined. Ground PLA is mixed with lignin and extruded at 180–190 °C. The addition of biopolymer improves melting properties and decreases tensile strength (18%) and decreases Young’s modulus value by about 6% in comparison with samples made of pure PLA (Gkartzou et al. 2017). Carbon fibers were also used to strengthen the material. The recycled material had 25% higher bending strength compared with the original. The material recovery rate was equal to 100% CFR (carbon fiber reinforced) and 71% PLA respectively (Tian et al. 2017). This was the first upgrade of reused polymer properties. Constant degradation of physical properties of reused polymers indicated research on new adhesive reinforcement types. On this basis, dopamine was used, which is easily adsorbed on most surfaces. This property also allows the coating of polymers. Ground PLA is placed in an aqueous solution of dopamine with stirring for 4 h. After this time, PLA is dried and extruded. It was found that the mass distribution of PLA with dopamine starts already at 200 °C, when for pure PLA this value exceeds 320 °C. The coating of the material also increased the tensile strength by about 20% (Zhao et al. 2018a, b). Oxidizing stabilizers are used to improve the properties of recycled material. Hydroquinone and tropolone can play this role. Hydroquinone has been found to be a much better stabilizer, which captures free radicals and thus maintains the PLA chain length during thermal processing. Similar observations were found for the mechanical properties of the material (Pillin et al. 2008).

Otherwise, polyethylene terephthalate (PET) and post-pyrolysis packing waste (0.5% and 5% wt.) biochar filament was used as 3D printing material. Tensile strength increased significantly (32% and 60%, respectively) compared with the pure PET shred (Idrees et al. 2018). Likewise, the thermal and dimensional stability in the case of mixing compounds was improved. Possible application for new 3D printing of mats created on the lignocelluloses matrix (based on cellulose, hemicelluloses, and lignin) with recycled PET has been examined (Santos et al. 2018). Depending on orientation of mats (directional or oppositional) in the alignment of fibers, investigated mechanical properties had different values (for example tensile strength value for directional 15.72 MPa and for oppositional 2.5 MPa). Finally, the result disclosed potential application of mats for additive manufacturing.

A significant improvement of the mechanical properties of the recycled material was noted in the presence of an additive in the form of biocarbon. Used PET bottles were mixed with biocarbon (< 100 μm) and subjected to thermal treatment. The presence of an additional component increased the tensile strength of the material by 32%. An increase in modulus of elasticity (about 60%) and higher resistance to thermal and oxidizing conditions were determined (Idrees et al. 2018). Biocarbon is also used to reinforce other materials, including the natural origin of PLA. It was found that the additives, in combination with the additives of natural origin, improve the stiffness of the samples (8%) (Notta-Cuvier et al. 2014).

To increase values of recycled polypropylene (PP), filaments based on hemp or harakeke fibers or recycled gypsum (0–50 wt.) were separately added in the process of creating new types of mats. The best results were achieved for filaments made of harakeke fibbers (30 wt.; tensile strength 39 MPa, Young modulus 2.8 GPa). On the other hand, those materials have tendency to reduce their properties during printing processes (Stoof and Pickering 2018).

Another attempt of enhancing quality of recycling polymers was based on the idea of incorporating nano-crystalline powders Fe, Si, Cr, and Al into PP and HDPE filament extrusion. It was found that adding 1% mix of powder (Fe-Si-Cr or Fe-Si-Al) resulted in better yield strength (37%) and Young modulus (17%) compared with the base materials values. Metals also reduces chances of crack formations (Pan et al. 2018). A significant improvement in mechanical strength of recycled HDPE was also found by using an additive in the form of SiC/Al_2_O_3_. HDPE waste is mixed with reinforcement (SiC/Al_2_O_3_) with the addition of paraffin wax as a binding agent. The prepared mixture is introduced into a screw extruder. It was noted that the additive slightly affects the thermal parameters of the material but significantly increases its mechanical strength (Singh et al. 2018). In another study, Singh et al. (2019a) proposed the use of zirconium oxide as a recycled HDPE enhancement element. The additive is mechanically mixed with the polymer and then ground in a ball mill. The filament is then extruded at 190 °C. It was observed that the coefficient of friction for the unreinforced material is 40% higher than for the polymer with zirconium oxide. The new polymer can be used as a building material for low-temperature bearings.

Adding styrene-ethylene-butylene-styrene (SEBS) as a stabilization promoter to the PET, PP, and PE blend as feedstock for 3D printing was probed. The blends had worst tensile strength (23 MPa for PP/PS mix, 19 MPa after addition of SEBS ) than pure recycled PET (35 MPa). Glass transition in most cases was shifted into higher temperature range (except the 50–50 wt. % PP/PET trial) (Zander et al. 2019). The introduction of an additional component or several components significantly improved the properties of the recycled material. This is an innovative approach and a solution to the problem with a limited amount of material reuse.

### Degradation of polymers

The growing interest in 3D printing technology and simple operation of devices have contributed to the popularity of low-budget printers, and many users can create their 3D structures at home (Wojtyła et al. 2017). The market offers a wide range of filaments of various materials. The main components of the commercial filament are thermoplastics. During 3D printing, a phase change occurs in the filament material as a result of its heating (above the melting point), melting in the nozzle, and solidification after extrusion (Ding et al. 2019). Under the influence of elevated temperature, degradation of polymers can also occur that causes defects that are thermally unstable and can lead to structural changes due to depolymerization or random scission (Cao et al. 2004). Under the influence of elevated temperature, thermomechanical degradation may also include chain breaks, reducing molecular weight and viscosity. Even when using printouts, polymer degradation (photochemical, thermal, and hydrolytic factors) may occur. This affects the material properties and structure (Beltrán et al. 2018).

The assessment of the thermal stability of polymers is crucial. Through thermogravimetric analysis (TGA), changes in mass of a heated sample at a constant rate are monitored. This technique allows to determine the initial degradation temperature and to determine the thermal stability of the volatile components fractions of the tested polymer (Cao et al. 2004; Wojtyła et al. 2017) Thermal degradation studies were carried out on acrylonitrile butadiene styrene (ABS), polylactic acid (PLA), polyethylene terephthalate (PET) and nylon, which are commonly used in commercial filaments in 3D printers. The degradation temperatures of the ABS, PLA, PET, and nylon polymers are shown in Table 2.

**Table 2:** Degradation and printing temperatures for comercial ABS, PLA, PET, and nylon (Wojtyła et al. 2017)

Polymer	Printing temperature	Degradation temperature
Nylon	240–280 °C	390–450 °C
ABS	230–250 °C	380–430 °C
PLA	200–235 °C	300–400 °C
PET	160–210 °C	350–480 °C

The plastic recycling process is carried out in the presence of high temperatures. Determining the impact of recycling on the properties of polymers used for 3D printing is an important issue. It turns out that recycling with a washing stage results in a 20% decrease in PLA molecular weight (Beltrán et al. 2018). However, the degradation process had no significant effect on structure, degree of crystallinity, or even thermal stability after five extrusions (Beltrán et al. 2018). Pillin et al. (2008) conducted similar studies on the impact of seven injection cycles in the range of 175–190 °C on the properties of commercial PLA. A decrease in the glass transition temperature (from 66.2 °C to 56.5 °C for pure PLA and after 7 cycles, respectively) was noted, which could be associated with chain breakage. There was also a decrease in stress at break (from 66 MPa to 25 MPa), which is closely related to the reduction in chain length. Grinding or pre-treatment caused a decrease in hardness of 15%. It is possible that water is a factor that activates degradation. Recycling filaments is a relatively new area of interest, but it is already known that any re-melting and re-extrusion of the filament lead to greater polymer degradation. The effects of decomposition are changes in thermal stability, decrease in molecular weight, and deterioration of mechanical properties of polymers. These changes do not differ much from those in the materials that are not recycled. Thus, plastic waste and unsuccessful prints can be re-melted and processed for reuse in 3D printing (Zenkiewicz et al. 2009).

## Commercial applications of filaments made from recycled plastics

Nowadays, market offers various filaments made from recycled PLA, PET, glycol-modified PET (PET-G), ABS, and HIPS (Table 3). PET and PLA filaments are made from waste food containers and bottles, ABS filaments originate from car dashboards, and HIPS from refrigerators or automotive parts. An interesting resource for filament production may be nylon from fishy nets (Porthcurno) or thermoplastic urethane from recycled ski boots (CREAMELT® TPU-R). It should be noted that the proportion of recycled material is variable. It depends mainly on the producers and the technology and its parameters, as well as the raw material used to produce r-filament (Pakkanen et al. 2017).

**Table 3:** Commercial filaments made from recycled polymers

Manufacturer	Material	Source	Contnet of r-plastic (%)	Price (€)	Web adress
B-PET	PET	PET bottles	100%	n.a.	(www.bpetfilament.com; accessed 2020.04.08)
Filamentive	PLA	Factory waste streams	55%	35.57	(www.filamentive.com; accessed 2020.04.09)
	ASA	n.a.	50%	36.82	
	PETg	Plastic food containers and drinks bottles	99.5%	36.93	
	PET	Plastic bottles	100%	37.62	
	ABS	n.a.	64%	35.57	
Fila-cycle	PLA	Yogurt pots	100%	n.a.	(www.fila-cycle.com; accessed 2020.04.09)
	ABS	Automotive waste	n.a.		
	PET	Bottle plastics			
	HIPS	Automotive Industrial Plastics, Home Electronics Industry			
Refil	HIPS	Refrigerators	100%	n.a.	(www.re-filament.com; accessed 2020.04.09)
	ABS	Car dashboards			
	PLA	Food packaging			
	PET	Blue bottles			
Innofil3D	PET	Recycled PET materials	100%	39.95	(www.ultrafusefff.com; accessed 2020.04.09)
Fishy filaments Porthcurno	Nylon	Fishy nets	100%	34.21	(www.fishyfilaments.com; accessed 2020.04.09)
Tridea	PET	PET food containers	100%	29.99	(www.tridea.co; accessed 2020.04.09)
	PLA	n.a		27.99	
CREAMELT	TPU	Recycled ski boots	100%	37.43	(www.ceramelt.com; accessed 2020.04.09)

*n.a.* not available

A series of devices enabling extrusion of filaments from waste plastics is commercially available in many variants (Table 4). The filament extruder, available to every user, in addition to the standard functions allowing for the production of a granulate filament, also has a built-in grinder that allows the processing of any plastic material. The use of extruders is recommended for all users of 3D printers, as they generate high savings of up to 80%. Due to the good performance of extruders available on the market, they often provide users with self-sufficiency. Moreover, due to their small size, low price, and simple operation, they do not require any specialist knowledge and facilities to operate them. The versatility of 3D printers and extruders allows users to customize the film obtained from recycled plastic to their printer model and can change waste into feedstock (Pakkanen et al. 2017).

**Table 4:** Commercial desktop filament extruders

Manufacturer	Diameter tolerance	Filament diameter	Extrusion speed (m/min)	Grinder	Price (€)	Web address
ProtoCycler+	+/− 0.05 mm	n.a	3.05	Optional	1560.00	(www.redetec.com; accessed 2020.04.08)
Felfil	+/− 0.07 mm	1.75 or 2.85	1.15	No	719.00	( www.felfil.com; accessed 2020.04.08)
Filabot	n.a	1.75 or 2.85	6.35	No	2486.27	(“www.filabot.com; accessed 2020.04.08)
FilaFab	n.a	1.75 or 2.85	1*	No	849.86	(www.d3dinnovations.com; accessed 2020.04.08)
Filastruder	+/− 0.03 mm	1.75	0.91	No	276.34	(www.filastruder.com; accessed 2020.04.09)
Noztek	+/− 0.04 mm	1.75 or 3	2.5	No	1135.03	(www.noztek.com; accessed 2020.04.09)
Strooder	+/− 0.1 mm	1.75, 2.85, 3.00	1.5	No	1093,96	(www.omnidynamics.co.uk; accessed 2020.04.09)
3devo	+/− 0.05 mm	0.5–3.0	n.a	Optional	5350.00	(www.3devo.com; accessed 2020.04.09)

*kg/h

The processing of plastics and the production of 3D printer filaments is in line with the principles of the circular economy and significantly affects the environment by reducing anthropogenic contamination with plastics. Therefore, many educational projects aimed at organizing interactive events and spreading knowledge about plastic recycling are organized. The Perpetual Plastic Project aims to popularize knowledge about the utility and value of plastics. The organizers, in front of the event participants, convert plastic waste into new useful products using 3D printing technology. This is done through four stages: washing and drying of plastic waste, shredding, filament forming, and 3D printing. The whole process takes about 30 min (www.perpetualplasticproject.com; accessed 2020.04.09). “Go green, print like a dream” is the guiding principle of the Reflow project. Its aim is to transform PET bottles into an ecological rPET filament that can be successfully used in 3D printing. The organizers point out the need to process beverage bottles, as currently only 5% of the plastic produced is recycled. It is estimated that after 2050 there will be more plastic in the world’s seas and oceans than fish, leading to the extinction of the marine ecosystem (www.reflowfilament.com; accessed 2020.04.09). CocaCola® Company, the world’s leading PET bottle manufacturer, has also been involved in the fight against plastic waste. The EKOCYCLE Cube 3D Printer Project aims to recycle plastic waste using technology, design, and styling and create changes in the entire culture. The circular economy of plastic, and PET in particular, protects the planet from excessive waste. As a result of the project, rPET is produced, with properties identical to those of classic non-recycling PET (www.3dsystems.com; accessed 2020.04.09). Nefilatek is a Canadian startup, aiming to produce a HIPS and PC filament, based on 100% plastic recycling. The company is currently undergoing pre-implementation research and announces the commercialization of its products (www.nefilatek.com; accessed 2020.04.09). Also, global communities are formed, bringing together people working on solving the problem of plastic waste (i.e., Precious Plastic, Plastic Bank). Precious Plastic is a community that aims to promote and learn about recycling. In their extensive activity, they spread knowledge about recycling methods, presenting the applied solutions: machines (shredder, extruder, sheetpress), products (furniture, jewelry, interior design elements, as well as even construction materials), business tools (they teach how to earn money from recycling), bazaar (where to sell products), as well as offer a platform for the recycling community to exchange experiences and exchange information about organized events. Plastic Bank is considered to be the best known and recognized activity reducing the amount of plastic deposited in the seas and oceans. Since its inception (2013), the organization has recovered and recycled 6.25 million kilograms of plastics from sea water, including nearly 500 million 0.5-L bottles, more than 1.5 billion coffee cup lids, and over 500 billion plastic straws. The action involves 4300 collectors from Haiti, the Philippines, and Indonesia (www.plasticbank.com; accessed 2020.04.09). Recycle Rebuilt is an organization that aims to turn waste into an opportunity. It helps to learn how to recycle and use appropriate technologies and machines so that plastic waste can be turned into useful objects. Within the scope of its activities, it also makes available supplies and learns how to operate machines used during recycling. An example of Recycle Rebuilt operation is the action carried out on the island of Dominica, destroyed by a hurricane in 2017, whose huge problem is overcrowded landfill sites, threatening human health and the safety of ecosystems. The organization created a center for collecting, sorting, and processing plastics into everyday consumables (www.recyclerebuild.org; accessed 2020.04.09).

## Future perspectives and application

Plastics have gained tremendous popularity in the last years due to low production costs, light weight, durability, and high strength. But on the other hand those are very persistent materials, noxious for the environment, and non-biodegradable (Singh et al. 2016). Plastics have been popularly used for the last 50 years because of durability, versatility, and low production cost of these materials (Zhong and Pearce 2018). Annual global production accounts for over 320 million Mg, and it is estimated to reach 850 million Mg by the year 2050 (Zhong and Pearce 2018). During the last 30 years, the production of plastics increased five times. Along with very intense production, there is a problem of increasing amount of plastic wastes which calls for elaboration of environmentally friendly methods of waste plastic valorization (Zhong and Pearce 2018).

Conventional recycling incorporates collection and separation of plastics that are low-density materials. This poses emission by transportation. Another approach—distributed recycling—that relies on recycling of consumer’s own wastes by upcycling plastic wastes into filament of 3D printing in recyclebot is more environmentally friendly. This is a new concept of recycling on site of waste generation that is used as resource to make useful products (Zhong and Pearce 2018).

There are different ways of valorization of plastic wastes. It is possible to recycle plastics in terms of chemical, mechanical, energy, and re-extrusion. The most recommended are palletization and extrusion processes (Singh et al. 2016). Recycling of plastics is determined by the market needs (Stoof and Pickering 2018). However, there are some drawbacks of conventional recycling, such as disposal of wastes and emissions. In this case, closed-loop recycling by 3D printing would be promising (Zhao et al. 2018a, b). 3D printing is also called additive manufacturing (AM) that enables to obtain products that are individually customized (Zhao et al. 2018a). AM makes it possible to recover thermoplastics that can be utilized as feedstock for AM (Stoof and Pickering 2018). The potential and prospective directions of application are medical, food, construction materials, toys, etc. (Zhao et al. 2018a, b).

Additive manufacturing in polymers recycling can proceed with simultaneous enhancement of materials’ thermal, mechanical, and tribological properties by forming composites that constitute polymeric matrix that is reinforced with fiber, ceramics, metal, or glass (Boparai et al. 2016). Such improved materials require characterization by different techniques to investigate also degradation of the material: thermal analysis (DSC), thermogravimetric analysis (TGA), dynamic mechanical analysis (DMA), Fourier-transform infrared spectroscopy (FT-IR), as well as thermal conductivity (Boparai et al. 2016). Thermoplastic polymers can also be used to obtain composite materials that add value by improving esthetic and mechanical characteristic of the material (Stoof and Pickering 2018). 3D printing enables to produce at home value-added products with complicated geometries. So-called recyclebots—extruders of waste plastic for the production of 3D printer filaments from the recycled plastic—seem to be useful (Hunt et al. 2015). Different materials can be used, e.g., polylactic acid (PLA) or acrylonitrile butadiene styrene (ABS) from spent materials (Hunt et al. 2015).

**Fig. 3:**
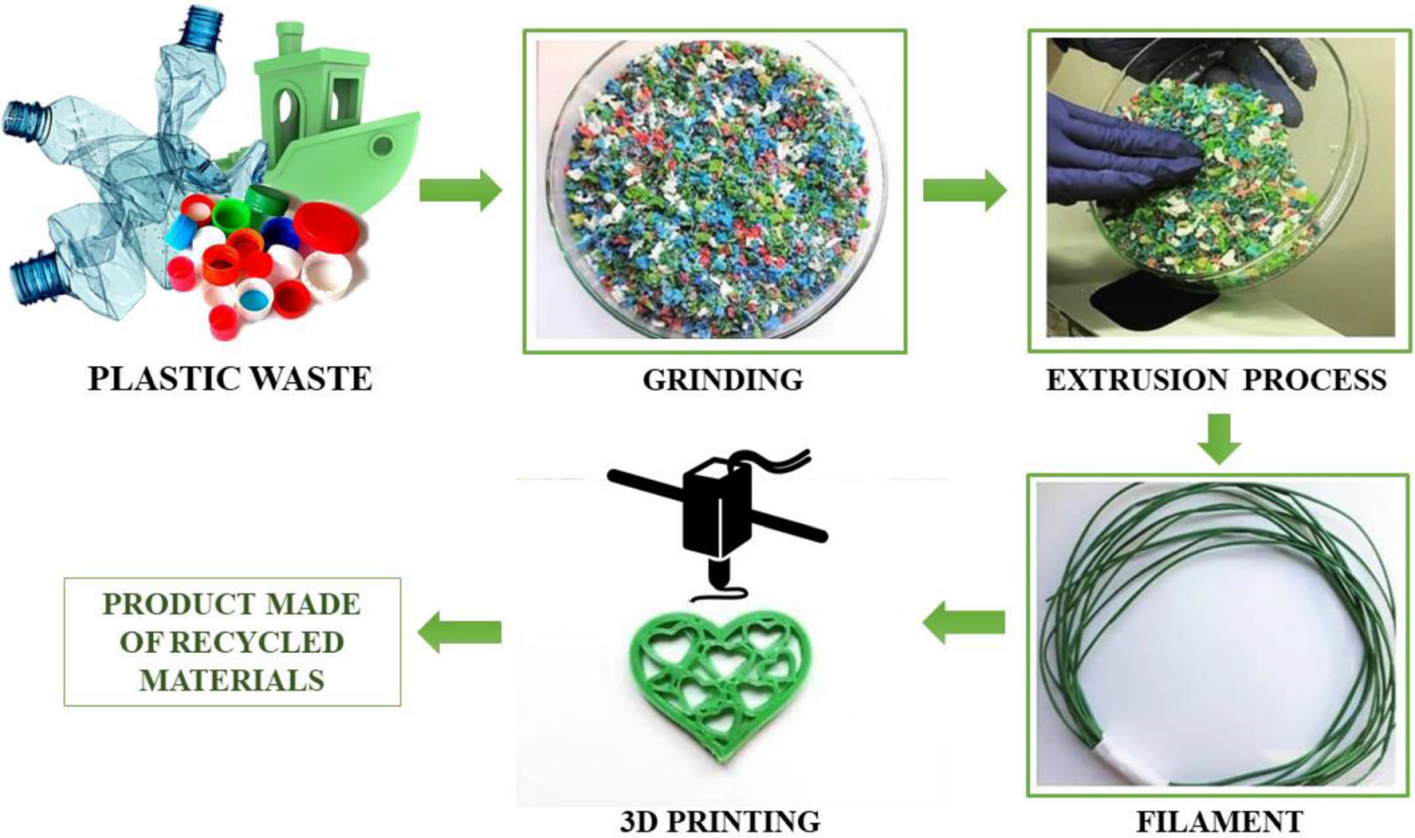
Recycling scheme for 3D printing materials

The reuse of unsuccessful prints, used parts, disposable prototypes, and waste materials not necessarily originally used for 3D printing, as a source of materials for filament production is beneficial both economically and for the environmental (Fig. 3). This reduces both material costs and CO2 emission and energy consumption. The required equipment, in the form of shredders or extruders, can be the minor equipment in small businesses, as well as a large installation for processing plastic waste. Manufacturers have already taken up this challenge and there are such filaments available on the market. However, the prices of such products are similar to conventional materials. Also very important is the consumer awareness of the “recycled is not worse” concept, which could be helped by information campaigns. Recycling plastic waste has a great potential and benefits, but requires investment and consumer knowledge.

## Conclusions

Plastics are not susceptible to biodegradation and their decomposition causes additional contamination of the environment. Recycling was found to be the most advantageous method to valorize post-consumer plastics that stays in line with a concept of circular economy. Degradation of plastics lasts from 10 to 450 years. From the historical point of view, recycling has been undertaken by the means of large centralized plants that produce commodities of low value. This is related with high costs of transportation. 3D printing enables different approaches. Desktop 3D printing makes it possible to produce complicated plastic products at home instead of in factory. It is estimated that the value of this sector will increase intensively the next years. The idea is that consumer can produce goods directly from his own used materials. This provides several savings: environmental and buying commercial plastic goods and enables to close the loop of circular economy.

Also recently, during coronavirus crisis, we observe individual applications that are possible for given needs. There are examples of 3D printing of, e.g., visors as a medical cover that protects eyes from coronavirus infection. By popularization of 3D printing, it is possible to adjust production to temporary needs.

## Abbreviations

ABS: Acrylonitrile butadiene styrene
AM: Additive manufacturing
ASA: Acrylonitrile styrene acrylate
CE: Circular economy
CRF: Carbon fiber reinforced
DMA: Dynamic mechanical analysis
DSC: Differential scanning calorimeter
FT-IR: Fourier-transform infrared spectroscopy
HDPE: High-density polyethylene
HIPS: High-impact polystyrene
LDPE: Low-density polyethylene
LLDPE: Linear low-density polyethylene
MAPP: Maleated polypropylene
PC: Polycarbonate
PCL: Polycaprolactone
PDA: Polydopamine
PE: Polyethylene
PEEK: Polyetheretherketone
PEI: Polyetherimide
PEMRG: Plastics Europe Market Research Group
PET: Polyethylene terephthalate
PETg: Polyethylene terephthalate glycol
PET-G: Glycol modified polyethylene terephthalate
PLA: Polylactic acid
PP: Polypropylene
PS: Polystyrene
PVC: Polyvinyl chloride
TGA: Thermogravimetric analysis
TPU: Thermoplastic polyurethane
VOC: Volatile Organic Compound
